# Effects of Licorice on clinical symptoms and laboratory signs in moderately ill patients with pneumonia from COVID-19: A structured summary of a study protocol for a randomized controlled trial

**DOI:** 10.1186/s13063-020-04706-3

**Published:** 2020-09-15

**Authors:** Omid Safa, Mehdi Hassani-Azad, Mehdi Farashahinejad, Parivash Davoodian, Habib Dadvand, Soheil Hassanipour, Mohammad Fathalipour

**Affiliations:** 1 Department of Clinical Pharmacy, Faculty of Pharmacy, Hormozgan University of Medical Sciences, Bandar Abbas, Iran; 2 Infectious and Tropical Diseases Research Center, Hormozgan Health Institute, Hormozgan University of Medical Sciences, Bandar Abbas, Iran; 3 Gastrointestinal and Liver Diseases Research Center, Guilan University of Medical Sciences, Rasht, Iran; 4 Department of Pharmacology and Toxicology, Faculty of Pharmacy, Hormozgan University of Medical Sciences, Bandar Abbas, Iran; 5 Endocrinology and Metabolic Research Center, Hormozgan University of Medical Sciences, Bandar Abbas, Iran

**Keywords:** COVID-19, Randomized controlled trial, Protocol, Liquorice, Clinical symptoms, Laboratory signs

## Abstract

**Objectives:**

We investigate the effects of Licorice (*Glycyrrhiza glabra* L.) root extract, an anti-inflammatory natural medicine, compared to the usual therapeutic regimen on clinical symptoms and laboratory signs in patients with confirmed COVID-19 that are moderately ill.

**Trial design:**

This is a single-center, open-label, randomized, clinical trial with parallel-group design. This study is being conducted at Shahid Mohammadi Hospital, Bandar Abbas, Iran.

**Participants:**

Both male and female patients with ≥18 years of age (≥ 35 kg of weight), admitted at the Shahid Mohammadi Hospital, Hormozgan University of Medical Sciences, Bandar Abbas for treatment, screened for the following criteria.

*Inclusion criteria*:

1. Confirmed diagnosis of SARS-CoV-2 infection (via polymerase chain reaction [PCR] and/or antibody test).

2. Presenting as moderate COVID-19 pneumonia (via chest computed tomography (CT) and/or X-ray) requiring hospitalization.

3. Hospitalized ≤48 hours.

4. Signing informed consent and willingness of study participant to accept randomization to any assigned treatment arm.

*Exclusion criteria*:

1. Underlying diseases, including chronic heart disease, chronic hypertension, severe renal failure, severe liver failure, and thyroid disorders.

2. Severe and critical COVID-19 pneumonia.

3. Use of warfarin, selective serotonin reuptake inhibitors (SSRIs), monoamine oxidase inhibitors (MAOIs), diuretics, corticosteroids, and antiarrhythmic drugs.

4. Treatment with Investigational and antiviral therapy in a clinical study within one month before randomization.

5. History of allergy to Licorice.

6. Pregnancy and breastfeeding.

**Intervention and comparator:**

*Intervention group*: The standard treatment regimen for COVID-19 along with a Licorice-based herbal preparation (D-Reglis ®, Irandarouk Pharmaceutical Company, Iran) at a dose of 760 mg three times a day for a period of seven days.

*Control group*: The standard treatment for COVID-19 based on the Iranian Ministry of Health and Medical Education's protocol for a period of seven days.

**Main outcomes:**

The recovery rate of clinical symptoms, including fever, dry cough, and tiredness, as well as paraclinical features, including thrombocytopenia, lymphocytopenia, and C-reactive protein, are evaluated as primary outcomes within seven days of randomization.

Time to improvement of clinical and paraclinical features and length of stay in a hospital, along with the incidence of adverse reactions are also evaluated as the secondary outcomes within seven days of randomization.

**Randomization:**

An electronic table of random numbers will be used to allocate the included participants into either control or intervention groups (in a 1:1 ratio) using the simple randomization method.

**Blinding (masking):**

This is an open-label trial without blinding and placebo control.

**Numbers to be randomized (sample size):**

A total of 60 participants randomizes (30 patients allocated to the intervention group and 30 patients allocated to the control group).

**Trial Status:**

The protocol is Version 1.0, May 31, 2020. Recruitment began July 30, 2020, and is anticipated to be completed by October 30, 2020.

**Trial registration:**

This clinical trial has been registered in the Iranian Registry of Clinical Trials (IRCT). The registration number is “IRCT20200506047323N2”, https://www.irct.ir/trial/47990. The registration date is 31 May 2020.

**Full protocol:**

The full protocol is attached as an additional file, accessible from the Trials website (Additional file 1). In the interest in expediting dissemination of this material, the familiar formatting has been eliminated; this Letter serves as a summary of the key elements of the full protocol.

## Supplementary information

**Additional file 1.** Full Study Protocol.

## Data Availability

The corresponding author has access to the final dataset of the trial, and the data will be available on reasonable request (Contact: M.fathalipour@hums.ac.ir).

