# Supplementary material for: Effects of Licorice on clinical symptoms and laboratory signs in moderately ill patients with pneumonia from COVID-19: A structured summary of a study protocol for a randomized controlled trial
Source: Trials. 2020 Sep 15;21:790. doi: 10.1186/s13063-020-04706-3 (PMC7490778; doi:10.1186/s13063-020-04706-3)
Supplement: Supplementary file 1 — Additional file 1. Full Study Protocol. [file 13063_2020_4706_MOESM1_ESM.pdf]

Protocol

This trial protocol has been provided by the authors to give readers additional information about their work.

Effects of Licorice on clinical symptoms and laboratory signs in patients with COVID-19: A structured summary of a study protocol for a randomized controlled trial

Omid Safa [1], Mehdi Hassani-Azad [2], Mehdi Farashahinejad [2], Parivash Davoodian [2], Habib Dadvand [2], Soheil Hassanipour [3], Mohammad Fathalipour [4,5]

[1] *Department of Clinical Pharmacy, Faculty of Pharmacy, Hormozgan University of Medical Sciences, Bandar Abbas, Iran.*

[2] *Infectious and Tropical Diseases Research Center, Hormozgan Health Institute, Hormozgan University of Medical Sciences, Bandar Abbas, Iran.*

[3] *Gastrointestinal and Liver Diseases Research Center, Guilan University of Medical Sciences, Rasht, Iran.*

[4] *Department of Pharmacology and Toxicology, Faculty of Pharmacy, Hormozgan University of Medical Sciences, Bandar Abbas, Iran.*

[5] *Endocrinology and Metabolic Research Center, Hormozgan University of Medical Sciences, Bandar Abbas, Iran.*

Corresponding author: Mohammad Fathalipour
Department of Pharmacology and Toxicology, Faculty of Pharmacy, Hormozgan University of Medical Sciences, Bandar Abbas, Iran.
Telephone: +98-9133962826
Fax: +98-7132307591
Email addresses: m.fathalipour@Hums.ac.ir

Abstract

Despite the large number of study carried out on the coronavirus disease 2019 (COVID-19), no effective pharmacological agents have yet been proven for the treatment of patients. Licorice (*Glycyrrhiza glabra* L.) is a strong demulcent that had been effectively used in the management of a number of diseases. The aim of the present study is to investigate the effects of Licorice root extract on clinical symptoms and laboratory signs in patients with covid-19.

A perspective open-label randomized clinical trial will be conducted on 60 hospitalized adult patients with covid-19 positive test (polymerase chain reaction test or chest CT-scan). Patients were randomly assigned in a 1:1 ratio to receive either Licorice root extract (760 mg three times a day) and standard care (lopinavir–ritonavir 200/50 mg twice a day and hydroxychloroquine 200 mg twice a day) for seven days, or standard care alone.

The primary outcomes are defined as recovery rate of clinical symptoms, including fever, high respiratory rate, low oxygen saturation, cough, myalgia, Headache, apnea, weakness, diarrhea, abdominal cramps, nausea, and vomiting. Secondary outcomes include the time from randomization to fever reduction and cough relief as well as the rate of oxygen therapy or noninvasive mechanical ventilation during the trial. Complete blood count and serum biochemical parameters will be checked on the first and seventh days. The adverse events and the diagnostic test will be evaluated at the end of study.

ارزیابی اثرات شیرین بیان بر علائم بالینی و یافته های آزمایشگاهی در بیماران مبتلا به COVID-19: یک کارآزمایی بالینی تصادفی برچسب باز

چکیده

با وجود تعداد زیادی مطالعه انجام شده در زمینه بیماری ناشی از ویروس کورونا (COVID-19)، هنوز هیچ داروی موثر دارویی برای درمان بیماران تایید نشده است. شیرین بیان (*Glycyrrhiza glabra* L.) یک تسکین دهنده بسیار موثر است که در درمان تعدادی از بیماری ها مورد استفاده قرار گرفته است. هدف از مطالعه حاضر، بررسی اثر عصاره ریشه شیرین بیان بر علائم بالینی و یافته های آزمایشگاهی در بیماران مبتلا به COVID-19 است.

این مطالعه به صورت یک کارآزمایی بالینی تصادفی با برچسب باز، روی ۶۰ بیمار بزرگسال با تست مثبت COVID-19 (تست PCR و یا Chest CT-scan) انجام می شود. بیماران به صورت تصادفی به نسبت ۱: ۱ در گروه دریافت کننده عصاره ریشه گیاه شیرین بیان (۷۶۰ میلی گرم سه بار در روز) و درمان استاندارد (لوپیناویر-ریتونوویر ۵۰/۲۰۰ میلی گرم دو بار در روز به همراه هیدروکسی کلروکین ۲۰۰ میلی گرم دو بار در روز) یا گروه دیافت کننده درمان استاندارد به تنهایی به مدت هفت روز، قرار خواهند گرفت.

پیامد های اولیه شامل میزان بهبودی علائم بالینی شامل تب، فرکانس تنفس بالا، اشباع کم اکسیژن شریانی، سرفه، درد عضلانی، سردرد، تنگی نفس، ضعف و بی حالی، اسهال، اسپاسم شکمی، تهوع و استفراغ می باشد. پیامد های ثانویه شامل فاصله زمانی شروع مطالعه تا کاهش تب و کاهش سرفه و همچنین میزاننیار به اکسیژن درمانی یا تهویه مکانیکی غیر تهاجمی در طول آزمایش خواهد بود. شمارش کامل سلول های خون و پارامترهای بیوشیمیایی سرم در روزهای اول و هفتم مطالعه بررسی می شود. عوارض جانبی و آزمایش تشخیصی در پایان مطالعه ارزیابی می شود.

مقدمه

در اواخر دسامبر سال ۲۰۱۹، یک بیماری تحت عنوان COVID-19[1] در اثر یک کروناویروس جدید که بعد ها SARS-CoV-2[2] نامیده شد، در ووهان چین پدیدار گردید و به سرعت در چین و خارج از آن شیوع یافت (۱, ۲). به دنبال شیوع این بیماری در بسیاری از کشور ها، سازمان بهداشت جهانی[3] در ۱۲ مارس سال ۲۰۲۰ اپیدمی COVID-19 را به عنوان پاندمی اعلام کرد (۳).

علائم شایع این بیماری شامل تب، سرفه و تنگی نفس است (۴). با این حال، علائم دیگری همچون خستگی، درد عضلات، گلو درد، اختلال در حس بویایی، اسهال و اسپاسم گوارشی ممکن است در بیماران بروز پیدا کند (۴-۶). علائم به طور معمول ۵ روز پس از تماس با ویروس شروع می شوند، اما این عدد ممکن است ۱۴-۲ روز متغیر باشد (۷). در حالی که اکثر بیماران دچار علائم خفیف می شوند، در برخی موارد این بیماری به پنمونی ویروسی[4] و نارسایی چند اندامی منجر می شود (۸). طبق مطالعاتی که اخیرا در چین انجام شده است، حدود ۸۰ درصد از بیماران مبتلا، بیماری خفیف را تجربه می کنند و میزان کلی مرگ و میر حدود ۲/۳ درصد است. اما در بیماران ۷۰ تا ۷۹ ساله این میزان به ۱۴/۸ درصد و در افراد بالای ۸۰ سال به ۸/۰ درصد افزایش می یابد (۹). در ۱۷ آوریل ۲۰۲۰، بیش از ۲/۱۶ میلیون مورد ابتلا به این بیماری در ۲۱۰ کشور گزارش شده است، که مرگ بیش از ۱۴۵۰۰۰ نفر را به همراه داشته است.

بر این اساس، تعداد چشم گیری از ناقلان بدون علامت در جمعیت وجود دارد، و بنابراین احتمالاً میزان مرگ و میر بیش از حد ارزیابی می شود. نهایتاً، یک درمان مؤثر برای معالجه بیماران علامت دار در جهت کاهش میزان مرگ و میر به ویژه در گروه های پرخطر، تخفیف علایم بیماری و کاهش احتمال انتقال ویروس در جامعه یک

[1] Coronavirus disease 2019
[2] Severe acute respiratory syndrome coronavirus 2
[3] World Health Organization
[4] Viral pneumonia

نیاز فوری قلمداد می شود. در حال حاضر تعداد زیادی کار آزمایی بالینی جهت بررسی اثرات داروهای قدیمی ضد ویروس، ضد انگل و ضد باکتری در حال انجام است، زیرا اطلاعات فراوانی در زمینه ایمنی، عوارض جانبی، بیواکی والانسی و تداخلات دارویی این دارو ها در دسترس است. علی رغم تلاش های فراوان در این زمینه تا کنون هیچ واکسن و یا رژیم دارویی در درمان این بیماری توسط سازمان های نظارتی دنیا تائید نشده است (۱۰, ۱۱).

در میان داروهای کاندید برای درمان بیماری COVID-19، استفاده از گیاهان دارویی که در طب سنتی هم جهت درمان بیماری های تنفسی استفاده می شود، یک استراتژی جالب است که مورد توجه محققین قرار گرفته است. شیرین بیان[1] (*Glycyrrhiza glabra* L.) گیاهی است که مصرف درمانی داشته و سابقه این مصرف به چند هزار سال قبل باز می گردد. این گیاه دارای اثرات مناسب در درمان مشکلات پوستی مانند درماتیت، اگزما و خارش می باشد و اثرات آنتی سپتیک خوب از آن گزارش شده است (۱۲). در طب سنتی ایران از این گیاه به عنوان درمان التهاب معده و سرفه استفاده می شود. این گیاه با توانایی مهار هلیکوباکترپیلوری، در درمان زخم های پپتیک و دوازدهه، گاستریت مزمن و بیماری های التهابی سیستم گوارش موثر می باشد. در مطالعات دیگر اثرات ضد تب، ضد التهاب، آنتی باکتریال و ضد ویروس این گیاه بررسی و به اثبات رسیده است. علاوه بر این شیرین بیان دارای خاصیت ضد سرفه، خلط آوری و ضد التهاب می باشد (۱۳) و در درمان عفونت های دستگاه تنفس فوقانی و برونشیت مورد استفاده قرار گرفته است (۱۴).

مطالعات بالینی متعددی بر عصاره ریشه شیرین بیان انجام شده است و ایمنی آن در تمامی مطالعات در دوز های مورد مطالعه به اثبات رسیده است. عصاره شیرین بیان به علت داشتن مقادیر زیاد گلیسیریزین می تواند موجب بروز عوارض مینرالوکورتیکوییدی به صورت احتباس آب و سدیم، دفع پتاسیم و افزایش فشار خون شود. اما در اکثر فراورده های تجاری موجود از شیرین بیان گلیسیریزین موجود در عصاره شیرین بیان با عمل هیـــدرولیز از۳۰٪ به کمتر از ۳٪ کاهش یافته است، که احتمال عوارض مینرالوکورتیکوییدی را بسیار کاهش می دهد. مصرف

[1] Liquorice

طولانی مدت این گیاه (بیش از ۶ ماه) ممکن است با حتمال بیشتری منجر به بروز دفع پتاسیم و افزایش فشار خون گردد, که با قطع مصرف به مدت ۲ هفته, این عوارض برطرف می شود. به صورت کلی توصیه می شود این گیاه در دوران بارداری و شیردهی، کودکان زیر ۶ سال، بیماران مبتلا به بیماری های قلبی عروقی, فشار خون بالا، هپاتیت مزمن, سیروز کبدی, بیماری های کلستاتیک کبد، التهاب کیسه صفرا, اختلالات شدید کلیوی و پرکاری غدد فوق کلیوی این گیاه را مصرف نکنند.

مطالعه حاضر با هدف بررسی اثر بخشی عصاره ریشه گیاه شیرین بیان در بیماران مبتلا به COVID-19 بستری شده در بیمارستان شهید محمدی شهرستان بندرعباس انجام خواهد شد.

بررسی متون

شیرین بیان گیاهی است که ریشه آن مصرف درمانی داشته و سابقه این مصرف به چند هزار سال قبل باز می گردد (۱۵) و یکی از معدود گیاهانی می باشد که تعداد زیادی مطالعه در خصوص ترکیبات و اثرات فارماکولوژیک آن انجام شده است (۱۶). عصاره گیاه شیرین بیان سال ها در طب سنتی چین بر ای مصارف درمانی مختلف مورد استفاده قرار گرفته است (۱۲). از این گیاه به فراوانی در داروسازی صنعتی به عنوان چسب فراورده های دارویی استفاده می شود.

از شناخته ترین خواص درمانی شیرین بیان، اثرات آن بر زخم معده می باشد، که مطالعات متعدد نشان داده اند فلاونوئید های موجود در ریشه این گیاه اثرات ضد هلیکوباکتری دارند (۱۷). گلیسیریزنیک اسید[1] موجود در این گیاه با توانایی مهار باکتری هلیکوباکترپیلوری، در درمان زخم معده، مشکلات مخاطی معده و کاهش اسید معده مؤثر است (۱۸, ۱۹). شیرین بیان از طریق مهار فسفودی استراز-۳[2] موجب اثرات ضد اسپاسمی گوارشی و تسکین درد احشایی می شود. عصاره هیدروالکلی آن از طریق افزایش فاکتورهای دفاعی مخاط معده موجب بروز فعالیت های ضد زخم معده می شود، بنابراین عصاره این گیاه برای درمان زخم معده و رفع اسپاسم مجاری گوارشی مفید است. شیرین بیان بر سیستم غدد درون ریز بدن تأثیر می گذارد و مصرف آن ممکن است مقدار تستوسترون خون را کاهش دهد و ثابت شده است که ریشه خشک شیرین بیان اثرات افزایش ترشح سرتونین و پروستاگلاندین در معده را دارد و اثرات ضد تورم معده را از این طریق اعمال می کند (۲۰).

علاوه بر این اثرات آنتی اکسیدان (۲۱) و آنتی موتاژنیک (۲۲) در مطالعات متعدد گزارش شده است. تأثیر ضد سرطانی روی سلول های ملانوم و سلول های سرطانی معده از طریق ایجاد آپپتوز می باشد. ترکیبات غیرپروتئینی موجود در ریشه گیاه شیرین بیان، ترکیبات اتانولی، فنولی، فلاونوئیدی و پلی ساکاریدی موجب آپوپتوز سلول می

[1] Glycyrrhetinic acid
[2] Phosphodiesterase 3

شود و می تواند از رشد سلول های سرطانی جلوگیری کند (۲۳). تحقیقات نشان داده است که عصاره اتانولی ریشه این گیاه، تقسیم سلولی در رده سلولی MCF-7 را مهار می کند و موجب جلوگیری از رشد این سلول ها در مرحله G1 و برانگیختن آپوپتوز در این سلول ها می گردد. فلاونوئید گیاه اثر ضد کارسینوژنیک دارد و باعث آپوپتوز در سلول های هپاتوما و ملانوما می شود و همچنین از رشد سلول های لوسمی مونوبلاستی جلوگیری می کند (۲۴, ۲۵). ترکیب ایزولیکورتیجنین[1] شیرین بیان با اثر حفاظتی موجب کاهش توان زیستی و افزایش آپوپتوز در سلول های سرطان پروستات می شود (۲۶). فلاونوئیدها و ترکیبات پلی فنولی گیاه جزء عوامل شیمی درمانی هستند که چرخه رشد سلول های توموری را در چند مرحله مورد تهاجم قرار می دهد.

مطالعات نشان می دهد عصاره ریشه شیرین بیان می تواند باعث مهار فعالیت آنزیم ۱۱-بتاهیدروکسی استروئید دهیدروژناز[2] شود و از تبدیل کورتیزول را به کورتیزون جلوگیری کند. همچنین از اشغال گیرنده های میترالوکورتیکوئیدی توسط استروئید های اندوژن در کلیه محافظت می نماید. بنابراین این گیاه اثرات کاهنده فعالیت گلوکوکورتیکوئیدی و مینرالوکورتیکوئیدی دارد (۲۷).

ریشه شیرین بیان در بیماران دیابتی می تواند برای درمان هیپرکالمی وابسته به هیپوآلدوسترونیسم بدون عارضه جانبی مورد استفاده قرار گرفته است (۲۸). فلاونوئیدهای هیدروفوبیک شیرین بیان چربی شکمی و قند خون را کاهش می دهد،که احتمالا این اثر از طریق فعالیت افزایشی گیرنده های گاماپراکسیزومی[3] اعمال می شود. علی رغم اینکه تاثیر شیرین بیان بر میزان قند خون در بیماران دیابتی چشم گیر نیست، اما میزان پرنوشی و پرخوری ناشی از دیابت را کم می کند (۲۹).

شیرین بیان به طور واضح سطوح کلسترول، LDL و تری گلیسرید خون را کاهش داده و HDL را افزایش می دهد و آسیب های آترواسکلروتیک شریانی را کم می کند. بنابراین شیرین بیان از پیشرفت آترواسکلروزیس جلوگیری

---

[1] Isoliquiritigenin
[2] 11β-Hydroxysteroid dehydrogenase
[3] Peroxisome proliferator-activated receptor gamma

می کند (۳۰, ۳۱). گلیسیریزین[۱] عمل ضد انعقادی دارد و از تشکیل ترومبین جلوگیری می کند و از تجمع پلاکت ها جلوگیری می کند (۳۲, ۳۳).

گلیدرینین[۲] موجود در عصاره این گیاه اثرات ضد تب و ضد التهاب دارد و به عنوان ایزوفلاون ریشه شیرین بیان دارای اثرات ضد میکروبی و محافظتی از قلب و عروق می باشد که این اثر از طریق مهار فاکتور NF-κB [۳] و در نتیجه مهار بیان ژن iNOS[۴] و تولید نیتریک اکساید صورت می گیرد (۳۴).

تأثیرات ضد ویروس این گیاه نیز علیه ویروس های متعددی ثابت شده است (۳۵). مطالعات نشان می دهد ترکیب گلیسیریزیک اسید[۵] در برابر طیف وسیعی از ویروس ها نظیر هرپس سیمپلکس[۶]، واریسلازوستر[۷]، سایتومگالوویروس[۸]، هپاتیت A، B، C و ویروس HIV[۹] موثر است (۳۶, ۳۷). گلیسیریزین و گلیسیریزنیک اسید به عنوان ترکیبات اساسی عامل اثرات ضد ویروس این گیاه باعث مهار اتصال و نفوذ ویروس به سلول میزبان در مراحل اولیه چرخه تکثیر می شوند. علاوه بر این، گلیسیریزین دارای خاصیت تقویت سیستم ایمنی بسیار خوبی است و با فعال سازی تکثیر لنفوسیت های T، اثرات ضد ویروس این گیاه را تقویت می کند (۳۸). مطالعات متعددی همچنین اثرات ضد ویروس این گیاه در درمان بیماری های آنفولانزا[۱۰] (۳۹) و سندرم تنفسی حاد[۱۱] (۳۸) را مشخص کرده اند. گلیسیریزین از طریق کاهش بار منفی در سطح سلولی و یا کاهش در ویسکوزیته غشای سلولی سبب مهار ورود ویروس هپاتیت به داخل سلول های کبدی می شود (۳۸). همچنین می تواند سبب کاهش ترنس آمینازهای[۱۲] سرم در بیماری هپاتیت C شود، اما به دنبال قطع مصرف آن ممکن است میزان آن ها مجددا

---

[1] Glycyrrhizin
[2] Glyderinine
[3] Nuclear factor kappa-light-chain-enhancer of activated B cells
[4] Inducible nitric oxide synthase
[5] Glycyrrhizic acid
[6] Herpes simplex viruses
[7] Varicella zoster virus
[8] Cytomegalovirus
[9] Human immunodeficiency viruses
[10] Influenza viruses
[11] Severe acute respiratory syndrome
[12] Serum transaminases

افزایش یابد (۴۰). همچنین نتایج امید بخشی از اثرات این گیاه در درمان ویروس HIV گزارش شده است (۴۱, ۴۲). در مطالعه ای که بر روی سوش های ویروس کرونا ایزوله شده از بیماران مبتلا به سندرم تنفسی حاد (SARS) انجام شده است، پتانسیل اثرات ضد ویروسی ترکیب گلیسیریزین بررسی گردید و مشخص شد که این ترکیب در مهار تکثیر ویروس در محیط های برون تنی بسیار موثر است (۴۳, ۴۴).

اثرات مفید متعددی نیز از گیاه شیرین بیان در کارآزمایی های بالینی به اثبات رسیده است. مطالعه بالینی انجام شده بر روی بیماران مبتلا به کبد چرب غیر الکلی[1]، نشان داده است که مصرف عصاره خشک گیاه شیرین بیان به میزان ۲ گرم در روز به مدت ۲ ماه باعث کاهش ترانس آمیناز های سرم در این افراد می شود، در حالی که عوارض ناخواسته ای بروز پیدا نمی کند (۴۵). افزودن عصاره خشک شیرین بیان به میزان ۳۸۰ میلی گرم ۲ بار در روز به رژیم استاندارد درمان هلیکوباکتر پیلوری کارایی این رژیم را به صورت معنی داری افزایش می دهد (۴۶). نتایج کارآزمایی که بر روی بیماران مبتلا به آلزایمر انجام شده، نشان می دهد مصرف عصاره خشک ریشه این گیاه به میزان ۱۳۶ میلی گرم دو بار در روز به مدت ۶ ماه می تواند باعث بهبود علایم این بیماری را در مقایسه با گروه پلاسبو می شود، در حالی که عوارض جانبی جدی ایجاد نمی کند (۴۷). در مطالعه دیگر که بر روی بیماران مسن مبتلا به استئوآرتریت انجام شده است، مشخص گردید که مصرف روزانه ۳۰۰ میلی گرم از روغن حاوی فلاونوئید های گیاه شیرین بیان برای ۱۶ هفته باعث افزایش توده عضلانی و کاهش میزان چربی شکمی در این بیماران در مقایسه با گروه پلاسبو می شود (۴۸). علاوه بر این مصرف این فراوده به میزان ۳۰۰ میلی گرم در روز به مدت ۱۶ هفته در افراد مسن باعث بهبود حفظ تعادل در مقایسه با پلاسبو شده است (۴۹). در کار آزمایی بالینی دیگر مشخص شده است که مصرف عصاره خشک این گیاه به میزان ۱/۵ گرم در روز به مدت ۸ هفته در افراد مبتلا به بیماری چاقی، اثرات کاهش وزن چشم گیری در مقایسه با گروه پلاسبو ندارد (۵۰)، در حالی که این درمان به صورت واضح باعث بهبود پروفایل لیپیدی این بیماران می شود (۵۱). بررسی ها نشان داده است

[1] Non-alcoholic fatty liver disease

اثرات ضد درد و ضد التهاب این گیاه در علائم دیس منوره[1] موثر است. مصرف عصاره خشک این گیاه به میزان ۷۵۰ میلی گرم ۲ بار در روز به مدت پنج روز (از شروع سیکل قاعدگی) به صورت معنی داری باعث بهبود علائم دیس منوره در مقایسه با گروه کنترل (ایبوبروفن) می شود (۵۲). مطالعات متعدد دیگری نیز اثرات مفید این گیاه به صورت فراورده های موضعی برای درمان آتروفی واژن[2] در سنین یائسگی (۵۳)، التهاب لثه[3] (۵۴)، التهاب مخاط دهان ناشی از پرتو درمانی[4] (۵۵) خشکی دهان[5] (۵۶)، درماتیت آتوپیک (۵۷)، اریتما[6] (۵۸)، برص[7] (۵۹) و آفت[8] (۶۰) نشان داده است.

[1] Dysmenorrhea
[2] Vaginal atrophy
[3] Gingivitis
[4] Radiotherapy-induced oral mucositis
[5] Xerostomia
[6] Erythema
[7] Vitiligo
[8] Aphthous ulcers

اهداف کلی طرح:

ارزیابی اثرات شیرین بیان در بیماران مبتلا به COVID-19:

اهداف ویژه‌ی طرح:

ارزیابی اثرات شیرین بیان بر علائم بالینی در بیماران مبتلا به COVID-19

مقایسه اثر بخشی درمان با دارو های استاندارد و درمان با دارو های استاندارد به همراه شیرین بیان بر علائم بالینی در بیماران مبتلا به COVID-19

ارزیابی اثرات شیرین بیان بر یافته های آزمایشگاهی در بیماران مبتلا به COVID-19

مقایسه اثر بخشی درمان با دارو های استاندارد و درمان با دارو های استاندارد به همراه شیرین بیان بر یافته های آزمایشگاهی در بیماران مبتلا به COVID-19

اهداف کاربردی طرح:

با توجه به این که اثرات مفیدی نظیر اثرات ضد التهاب، ضد سرفه، ضد ویروس و ضد باکتری از عصاره ریشه شیرین بیان در مطالعات بالینی متعدد به اثبات رسیده است، می توان گفت در درمان کمکی با این گیاه امکان بهبود کارایی رژیم های درمانی موجود وجود دارد و در صورت حصول پاسخ دهی بهتر نسبت به درمان استاندارد می توان در آینده این گیاه جهت بهبود سریع تر علائم بالینی و یافته های آزمایشگاهی بیماران، به درمان استاندارد اضافه شود.

فرضیات یا سوالات پژوهش (باتوجه به اهداف طرح):

علائم بالینی بیماران مبتلا به COVID-19 در گروه مصرف کننده شیرین بیان نسبت به گروه کنترل بهتر و سریع تر بهبود می یابد.

یافته های آزمایشگاهی بیماران مبتلا به COVID-19 در گروه مصرف کننده شیرین بیان نسبت به گروه کنترل بهتر و سریع تر بهبود می یابد.

روش اجرای طرح

طراحی مطالعه و شرکت کنندگان

این مطالعه در قالب یک کارآزمایی بالینی تصادفی با برچسب باز[1] بر روی ۶۰ بیمار مبتلا به بیماری COVID-19 بستری شده در بخش سندرم حاد تنفسی بیمارستان شهید محمدی شهرستان بندرعباس، ایران طراحی شده است. بیماران از ۳۰ آوریل ۲۰۲۰ به صورت آینده نگر وارد مطالعه و پیگیری خواهند شد. با توجه به نسبت ۱:۱ بین گروه آزمایش (پرتکل استاندارد کشوری به همراه شیرین بیان) و گروه کنترل (پرتکل استاندارد کشوری)، بیماران به صورت تصادفی وارد هر یک از بازو های مداخله می شوند.

در شروع مطالعه بیماران بر اساس علائم بالینی و یافته های پاراکلینیکی در گروه های خفیف، متوسط، شدید و بحرانی تقسیم بندی می شوند (جدول ۱). معیارهای ورود بیماران شامل؛ (۱) سن ۱۸ سال یا بالاتر، (۲) رضایت آگاهانه و داوطلبانه، (۳) علائم بالینی اولیه و (۴) تشخیص قطعی بیماری COVID-19 از طریق تست PCR[2] (و یا Chest CT-scan[3]) که شدت بیماری در گروه های متوسط، شدید و یا بحرانی باشد، خواهند بود. تمامی بیماران با سابقه ای از (۱) بیماری های قلبی و عروقی، افزایش فشار خون، هپاتیت مزمن، سیروز کبدی، بیماری های کلستاتیک کبد، التهاب کیسه صفرا، اختلالات شدید کلیوی، پرکاری غدد فوق کلیوی، (۲) مصرف دارو هایی همچون وارفارین، مهارکننده های انتخابی بازجذب سروتونین، مهارکننده های مونوآمین اکسیداز، دیورتیک ها، کورتیکواستروئید ها، دارو های هرمونی و دارو های ضد آریتمی و (۳) زنان در دوران بارداری و شیردهی از مطالعه خارج می شوند.

در ابتدای کارآزمایی، مشخصات عمومی، مشخصات دموگرافیک و سوابق پزشکی بیماران با استفاده از پرسشنامه جمع آوری می شود. پس از ارائه توضیحات کافی و کسب رضایت آگاهانه کتبی از جانب بیمار یا بستگان درجه

[1] Open-label randomized clinical trial
[2] Polymerase Chain Reaction
[3] Chest Computed tomography scan

یک (در بیماران با سطح هوشیاری پایین یا زوال عقل)، بیماران با استفاده از روش تصادفی سازی بلوک ها دو گروه مساوی (گروه مداخله و گروه کنترل) تقسیم می شوند.

گروه های مداخله

گروه A بیمارانی هستند که درمان استاندارد بر اساس پروتکل تعیین شده از سوی وزارت بهداشت برای بیماری COVID-19 را دریافت می کنند. این درمان شامل دارو های هیدروکسی کلروکین (۲۰۰ میلی گرم دو بار در روز) و کلترا (ترکیبی از دارو های لوپیناویر ۲۰۰ میلی گرم و ریتوناویر ۵۰ میلی گرم دو بار در روز) به مدت ۷ روز می باشد. گروه B بیمارانی هستند که علاوه بر درمان استاندارد فوق الذکر، یک فراورده گیاهی مبتنی بر شیرین بیان در شکل دارویی قرص، با دوز ۷۶۰ میلی گرم سه بار در روز به مدت ۷ روز دریافت می کنند. در این مطالعه از قرص های د-رگلیس شرکت داروسازی ایران داروک استفاده می شود گه هر قرص حاوی ۳۸۰ میلی گرم عصاره خشک استاندارد شده ریشه شیرین بیان می باشد.

بررسی پیامد ها

پیامد های اولیه این مطالعه میزان بهبودی علائم بالینی در طی دوره مداخله نظر گرفته می شود. بهبود علائم بالینی به عنوان بهبود مداوم (بیشتر از ۷۲ ساعت) درجه حرارت بدن، فرکانس تنفس و میزان اشباع اکسیژن خون پس از شروع درمان می باشد که با معیارهای کمی که در ادامه آمده است تعریف می شود: دمای دهانی ≤ ۳۶/۶ درجه سانتیگراد؛ فرکانس تنفسی ≤۲۴ بار در دقیقه و اشباع اکسیژن ≥۹۸ درصد بدون تنفس مکانیکی. علاوه بر این، نیاز به اکسیژن درمانی و تهویه با فشار مثبت غیر تهاجمی در فالوآپ های روزانه به همراه علائم بالینی دیگر علائم بالینی دیگر همچون سرفه، درد عضلانی، سردرد، تنگی نفس، ضعف و بی حالی، کاهش حس بویایی و چشایی، اسهال، اسپاسم شکمی، تهوع و استفراغ به صورت کیفی ثبت می شوند. اندازه گیری مکرر حداقل برای دو بار در هر فالوآپ انجام می شود.

پیامد های ثانویه شامل مدت زمان بستری بودن در بیمارستان، فاصله زمانی از شروع مطالعه (تصادفی سازی در هر یک از گروه های مطالعه) تا کاهش تب (بیماران مبتلا به تب)، فاصله زمانی از شروع مطالعه تا تسکین سرفه (بیماران مبتلا به سرفه متوسط یا شدید در زمان ثبت نام)، فاصله زمانی از شروع مطالعه تا بهبود تنگی نفس، میزان نیاز اکسیژن درمانی کمکی یا تهویه مکانیکی غیر تهاجمی در طول مطالعه، نیاز به بستری شدن در بخش مراقبت های ویژه و میزان نارسایی تنفسی در طول مطالعه (اشباع اکسیژن ≤۹۰ درصد بدون تنفس مکانیکی و یا PaO_2/FiO_2 >۳۰۰ میلی متر جیوه، نیاز به اکسیژن درمانی و یا حمایت تنفسی) می باشند.

انجام آزمایش های CBC[1]، شمارش تفریقی سلول های سفید، میزان فریتین، CRP[2]، LDH[3]، ESR[4]، کراتینین و نیتروژن اوره سرم در ابتدای مطالعه و انتهای مطالعه (روز ۷ مطالعه و یا زمان ترخیص) بررسی خواهد شد. علاوه بر این تست PCR (و یا Chest CT-scan) و انتهای مطالعه (روز ۷ مطالعه و یا زمان ترخیص) بررسی خواهد شد. همچنین واکنش های نا خواسته (به ویژه افزایش افزایش فشار خون)، فراوانی عوارض جانبی احتمالی ناشی از مداخله و فراوانی انصراف از مطالعه به علت عوارض جانبی در گروه های مورد مطالعه به صورت روزانه ثبت می شوند.

روش محاسبه حجم نمونه

با استفاده از مطالعات مشابه انجام شده در گروه های چند دارویی میزان بهبودی بالینی مورد انتظار در روز ۷ از گروه مورد مطالعه ۸۳ درصد، بهبودی بالینی گروه کنترل ۳۵ درصد، $\alpha = 0.05$، $\beta = 0.10$، قدرت = ۰/۹۰ در نظر گرفته شد. با توجه به توزیع ۱: ۱ بین گروه مورد مطالعه و گروه کنترل، حجم نمونه آماری ۲۴ شرکت کننده

[1] Complete blood count
[2] C-reactive protein
[3] Lactate dehydrogenase
[4] Erythrocyte sedimentation rate

در هر گروه است. اندازه نمونه با توجه به عواملی مانند ریختن حدود ۲۰ درصد افزایش یافته است. این کارآزمایی شامل ۶۰ نفر (۳۰ نفر در هر گروه) می باشد.

$$N_1 = \left\{ z_{1-\alpha/2} * \sqrt{\bar{p} * \bar{q} * (1 + \frac{1}{k})} + z_{1-\beta} * \sqrt{p_1 * q_1 + (\frac{p_2 * q_2}{k})} \right\}^2 / \Delta^2$$

$$q_1 = 1 - p_1$$

$$q_2 = 1 - p_2$$

$$\bar{p} = \frac{p_1 + kp_2}{1 + K}$$

$$\bar{q} = 1 - \bar{p}$$

```
Estimated sample size for two-sample comparison of proportions

Test Ho: p1 = p2, where p1 is the proportion in population 1
                    and p2 is the proportion in population 2
Assumptions:

         alpha =   0.0500  (two-sided)
         power =   0.9000
            p1 =   0.8300
            p2 =   0.3500
         n2/n1 =   1.00

Estimated required sample sizes:

            n1 =       24
            n2 =       24
```

روش تصادفی سازی بلوک

در این روش تعداد افراد در هر یک از گروه های مطالعه در طول درمان با یکدیگر برابر است. با توجه به تعداد ۶۰ نفر افراد شرکت کننده (۳۰ نفر در هر گروه) و مدت زمان تقریبی ۱۰ هفته برای تکمیل ورود افراد به مطالعه، از ۱۰ بلوک ۶ تایی (در صورت وجود بیمار به تعداد کافی از ۵ بلوک ۱۲ تایی) استفاده خواهد شد (جدول ۳). روش کار در این نوع از تصادفی سازی شبیه به روش تصادفی سازی ساده است، تنها تعداد افراد در طول دوره درمان

در دو گروه مداخله و کنترل یکسان می باشد. تنها ایراد این روش مشخص شدن آخرین گروه در هر بلوک می باشد.

آنالیز آماری

برای تحلیل آماری از نرم افزار SPSS ورژن ۱۸/۰ استفاده می شود. برای مقایسه شاخص های اصلی اثر بخشی (میزان بهبود علائم بالینی) به عنوان پیامد اولیه و پیامد های ثانویه بین گروه مورد مطالعه و گروه کنترل، از آزمون t برای متغیرهای پیوسته و یا آزمون Wilcoxon (در صورت عدم استفاده از آزمون t) برای متغییر های گسسته رتبه ای استفاده می شود. توصیف آماری متغیرهای کیفی به صورت فراوانی یا درصد مشاهده خواهد بود و برای مقایسه بین گروه ها از آزمون های Chi-square یا Fisher’s exact استفاده خواهد شد. برای کلیه آزمون های آماری، P <0.05 (دو طرفه) از نظر آماری معنی دار در نظر گرفته می شود.

ملاحظات اخلاقی

جهت شرکت در این مطالعه از تمامی بیماران رضایت آگاهانه اخذ خواهد شد. اطلاعات مربوطه بصورت محرمانه حفظ خواهد شد. هیچ یک از مشخصات فردی افراد شرکت کننده از جمله اسم و فامیل آنها وارد کامپیوتر نخواهد شد و به تمامی افراد کد پروژه داده شده و آنالیز بر اساس آن انجام خواهد شد. اطلاعات اولیه در فایل های قفل دار و نزد مجری اصلی تا اتمام پروژه و انتشار مقالات باقی خواهد ماند. در هر مرحله از طرح بیماران می توانند بنا به تمایل شخصی از طرح خارج شوند.

محدودیت‌های اجرایی طرح و روش رفع آن ها

عدم همکاری بیماران که امید است با توضیحات کافی برای آن ها این مشکل رفع شود و قبل از شروع به بیماران در مورد عوارض جانبی بیماری و دارو توضیح داده می شود. از آنها قبل از شروع درمان رضایت نامه کتبی دریافت می شود. همچنین این پژوهش از نظر اخلاق پزشکی مورد تایید است.

References

1. Lai C-C, Shih T-P, Ko W-C, Tang H-J, Hsueh P-R. Severe acute respiratory syndrome coronavirus 2 (SARS-CoV-2) and corona virus disease-2019 (COVID-19): the epidemic and the challenges. International journal of antimicrobial agents. 2020:105924.
2. Wang L-s, Wang Y-r, Ye D-w, Liu Q-q. A review of the 2019 Novel Coronavirus (COVID-19) based on current evidence. International Journal of Antimicrobial Agents. 2020:105948.
3. Organization WH. WHO Director-General's opening remarks at the media briefing on COVID-19-11 March 2020. Geneva, Switzerland. 2020.
4. Kailas Khandu Sanap D, Sanap AK. What We Know So Far About New Coronavirus (COVID-19). Sustainable Humanosphere. 2020;16(1):1470-6.
5. Magdi H. COVID-19 Coronavirus Disease. 2020.
6. Hopkins C, Kumar N. Loss of sense of smell as marker of COVID-19 infection. ENT UK at The Royal College of Surgeons of England. 2020.
7. Velavan TP, Meyer CG. The COVID-19 epidemic. Trop Med Int Health. 2020;25(3):278-80.
8. Hui DS, Azhar EI, Madani TA, Ntoumi F, Kock R, Dar O, et al. The continuing 2019-nCoV epidemic threat of novel coronaviruses to global health—The latest 2019 novel coronavirus outbreak in Wuhan, China. International Journal of Infectious Diseases. 2020;91:264.
9. Wu Z, McGoogan JM. Characteristics of and important lessons from the coronavirus disease 2019 (COVID-19) outbreak in China: summary of a report of 72 314 cases from the Chinese Center for Disease Control and Prevention. Jama. 2020.
10. Colson P, Rolain J-M, Lagier J-C, Brouqui P, Raoult D. Chloroquine and hydroxychloroquine as available weapons to fight COVID-19. Int J Antimicrob Agents. 2020;105932(10.1016).
11. Colson P, Rolain J-M, Raoult D. Chloroquine for the 2019 novel coronavirus. Int J Antimicrob Agents. 2020.
12. Wang X, Zhang H, Chen L, Shan L, Fan G, Gao X. Liquorice, a unique "guide drug" of traditional Chinese medicine: a review of its role in drug interactions. Journal of ethnopharmacology. 2013;150(3):781-90.
13. Kuang Y, Li B, Fan J, Qiao X, Ye M. Antitussive and expectorant activities of licorice and its major compounds. Bioorganic & Medicinal Chemistry. 2018;26(1):278-84.
14. Pastorino G, Cornara L, Soares S, Rodrigues F, Oliveira MBP. Liquorice (Glycyrrhiza glabra): A phytochemical and pharmacological review. Phytotherapy research. 2018;32(12):2323-39.
15. Mattarello MJ, Benedini S, Fiore C, Camozzi V, Sartorato P, Luisetto G, et al. Effect of licorice on PTH levels in healthy women. Steroids. 2006;71(5):403-8.
16. Asl MN, Hosseinzadeh H. Review of pharmacological effects of Glycyrrhiza sp. and its bioactive compounds. Phytotherapy Research: An International Journal Devoted to Pharmacological and Toxicological Evaluation of Natural Product Derivatives. 2008;22(6):709-24.
17. Khayyal MT, El-Ghazaly MA, Kenawy SA, Seif-El-Nasr M, Mahran LG, Kafafi YA, et al. Antiulcerogenic effect of some gastrointestinally acting plant extracts and their combination. Arzneimittelforschung. 2001;51(07):545-53.

18. Haraguchi H, Tanimoto K, Tamura Y, Mizutani K, Kinoshita T. Mode of antibacterial action of retrochalcones from Glycyrrhiza inflata. Phytochemistry. 1998;48(1):125-9.
19. Fukai T, Marumo A, Kaitou K, Kanda T, Terada S, Nomura T. Anti-Helicobacter pylori flavonoids from licorice extract. Life sciences. 2002;71(12):1449-63.
20. Jalilzadeh-Amin G, Najarnezhad V, Anassori E, Mostafavi M, Keshipour H. Antiulcer properties of Glycyrrhiza glabra L. extract on experimental models of gastric ulcer in mice. Iranian journal of pharmaceutical research: IJPR. 2015;14(4):1163.
21. Oganesyan K. Antioxidant effect of licorice root on blood catalase activity in vibration stress. Bulletin of experimental biology and medicine. 2002;134(2):135-6.
22. Alekperov UK. Plant antimutagens and their mixtures in inhibition of genotoxic effects of xenobiotics and aging processes. European journal of cancer prevention: the official journal of the European Cancer Prevention Organisation (ECP). 2002;11:S8-11.
23. SHIMIZU N, TOMODA M, TAKADA K, Gonda R. The core structure and immunological activities of glycyrrhizan UA, the main polysaccharide from the root of Glycyrrhiza uralensis. Chemical and pharmaceutical bulletin. 1992;40(8):2125-8.
24. Rossi T, Benassi L, Magnoni C, RUBERTO AI, COPPI A, BAGGIO G. Effects of glycyrrhizin on UVB-irradiated melanoma cells. in vivo. 2005;19(1):319-22.
25. Hsu Y-L, Kuo P-L, Lin L-T, Lin C-C. Isoliquiritigenin inhibits cell proliferation and induces apoptosis in human hepatoma cells. Planta medica. 2005;71(02):130-4.
26. Jung JI, Lim SS, Choi HJ, Cho HJ, Shin H-K, Kim EJ, et al. Isoliquiritigenin induces apoptosis by depolarizing mitochondrial membranes in prostate cancer cells. The Journal of nutritional biochemistry. 2006;17(10):689-96.
27. Al-Qarawi A, Abdel-Rahman H, Ali B, El Mougy S. Liquorice (Glycyrrhiza glabra) and the adrenal-kidney-pituitary axis in rats. Food and chemical toxicology. 2002;40(10):1525-7.
28. Murakami T, Uchikawa T. Effect of glycyrrhizine on hyperkalemia due to hyporeninemic hypoaldosteronism in diabetes mellitus. Life sciences. 1993;53(5):PL63-PL8.
29. Swanston-Flatt S, Day C, Bailey C, Flatt P. Traditional plant treatments for diabetes. Studies in normal and streptozotocin diabetic mice. Diabetologia. 1990;33(8):462-4.
30. Visavadiya NP, Narasimhacharya AV. Hypocholesterolaemic and antioxidant effects of Glycyrrhiza glabra (Linn) in rats. Molecular nutrition & food research. 2006;50(11):1080-6.
31. Ajay M, Achike FI, Mustafa MR. Modulation of vascular reactivity in normal, hypertensive and diabetic rat aortae by a non-antioxidant flavonoid. Pharmacological research. 2007;55(5):385-91.
32. Mendes-Silva W, Assafim M, Ruta B, Monteiro RQ, Guimarães JA, Zingali RB. Antithrombotic effect of Glycyrrhizin, a plant-derived thrombin inhibitor. Thrombosis research. 2003;112(1-2):93-8.
33. Mauricio I, Francischetti B, Monteiro RQ, Guimarães JA. Identification of glycyrrhizin as a thrombin inhibitor. Biochemical and biophysical research communications. 1997;235(1):259-63.
34. Azimov M, Zakirov U, Radzhapova S. Pharmacological study of the anti-inflammatory agent glyderinine. Farmakologiia i toksikologiia. 1988;51(4):90-3.
35. Wang L, Yang R, Yuan B, Liu Y, Liu C. The antiviral and antimicrobial activities of licorice, a widely-used Chinese herb. Acta Pharmaceutica Sinica B. 2015;5(4):310-5.

36. Baba M, Shigeta S. Antiviral activity of glycyrrhizin against varicella-zoster virus in vitro. Antiviral research. 1987;7(2):99-107.
37. Sato H, Goto W, Yamamura J-i, Kurokawa M, Kageyama S, Takahara T, et al. Therapeutic basis of glycyrrhizin on chronic hepatitis B. Antiviral Research. 1996;30(2-3):171-7.
38. Soufy H, Yassein S, Ahmed AR, Khodier MH, Kutkat MA, Nasr SM, et al. Antiviral and immune stimulant activities of glycyrrhizin against duck hepatitis virus. African Journal of Traditional, Complementary and Alternative Medicines. 2012;9(3):389-95.
39. Utsunomiya T, Kobayashi M, Pollard RB, Suzuki F. Glycyrrhizin, an active component of licorice roots, reduces morbidity and mortality of mice infected with lethal doses of influenza virus. Antimicrobial Agents and Chemotherapy. 1997;41(3):551-6.
40. Arase Y, Ikeda K, Murashima N, Chayama K, Tsubota A, Koida I, et al. The long term efficacy of glycyrrhizin in chronic hepatitis C patients. Cancer. 1997;79(8):1494-500.
41. Ito M, Nakashima H, Baba M, Pauwels R, De Clercq E, Shigeta S, et al. Inhibitory effect of glycyrrhizin on the in vitro infectivity and cytopathic activity of the human immunodeficiency virus [HIV (HTLV-III/LAV)]. Antiviral research. 1987;7(3):127-37.
42. Sasaki H, Takei M, Kobayashi M, Pollard RB, Suzuki F. Effect of glycyrrhizin, an active component of licorice roots, on HIV replication in cultures of peripheral blood mononuclear cells from HIV-seropositive patients. Pathobiology. 2002;70(4):229-36.
43. Cinatl J, Morgenstern B, Bauer G, Chandra P, Rabenau H, Doerr H. Glycyrrhizin, an active component of liquorice roots, and replication of SARS-associated coronavirus. The Lancet. 2003;361(9374):2045-6.
44. Nassiri Asl M, Hosseinzadeh H. Review of Antiviral Effects of Glycyrrhiza glabra L. and Its Active Component, Glycyrrhizin. jmpir. 2007;2(22):1-12.
45. Hajiaghamohammadi AA, Ziaee A, Samimi R. The efficacy of licorice root extract in decreasing transaminase activities in non-alcoholic fatty liver disease: A randomized controlled clinical trial. Phytotherapy Research. 2012;26(9):1381-4.
46. Hajiaghamohammadi AA, Zargar A, Oveisi S, Samimi R, Reisian S. To evaluate of the effect of adding licorice to the standard treatment regimen of Helicobacter pylori. The Brazilian Journal of Infectious Diseases. 2016;20(6):534-8.
47. Petramfar P, Hajari F, Yousefi G, Azadi S, Hamedi A. Efficacy of oral administration of licorice as an adjunct therapy on improving the symptoms of patients with Parkinson's disease, A randomized double blinded clinical trial. Journal of Ethnopharmacology. 2020;247.
48. Kinoshita T, Matsumoto A, Yoshino K, Furukawa S. The effects of licorice flavonoid oil with respect to increasing muscle mass: a randomized, double-blind, placebo-controlled trial. Journal of the Science of Food and Agriculture. 2017;97(8):2339-45.
49. Kinoshita T, Maruyama K, Yamamoto N, Saito I. The effects of dietary licorice flavonoid oil supplementation on body balance control in healthy middle-aged and older Japanese women undergoing a physical exercise intervention: a randomized, double-blind, placebo-controlled trial. Aging Clinical and Experimental Research. 2020.
50. Alizadeh M, Namazi N, Mirtaheri E, Sargheini N, Kheirouri S. Changes of insulin resistance and adipokines following supplementation with Glycyrrhiza glabra L. Extract in combination with a low-calorie diet in overweight and obese subjects: A randomized double blind clinical trial. Advanced Pharmaceutical Bulletin. 2018;8(1):123-30.

51. Mirtaheri E, Namazi N, Alizadeh M, Sargheini N, Karimi S. Effects of dried licorice extract with low-calorie diet on lipid profile and atherogenic indices in overweight and obese subjects: A randomized controlled clinical trial. European Journal of Integrative Medicine. 2015;7(3):287-93.
52. Jafari Z, Emtiazy M, Sohrabvand F, Talei D, Oveidzadeh L, Abrishamkar M, et al. The effect of Glycyrrhiza glabra L. On primary dysmenorrhea compared with ibuprofen: A randomized, triple-blind controlled trial. Iranian Journal of Pharmaceutical Research. 2019;18(Special Issue):291-301.
53. Sadeghi M, Namjouyan F, Cheraghian B, Abbaspoor Z. Impact of Glycyrrhiza glabra (licorice) vaginal cream on vaginal signs and symptoms of vaginal atrophy in postmenopausal women: A randomized double blind controlled trial. Journal of Traditional and Complementary Medicine. 2020;10(2):110-5.
54. Molania T, Saeedi M, Ehsani H, Khazaei Z, Moosazadeh M, Rostamkalaei S, et al. Evaluation of anti-inflammatory effect of oral licorice in comparison with chlorhexidine in population with gingivitis: A double blind clinical trial study. Gazi Medical Journal. 2019;30(2):144-9.
55. Ghalayani P, Emami H, Pakravan F, Nasr Isfahani M. Comparison of triamcinolone acetonide mucoadhesive film with licorice mucoadhesive film on radiotherapy-induced oral mucositis: A randomized double-blinded clinical trial. Asia-Pacific Journal of Clinical Oncology. 2017;13(2):e48-e56.
56. Yu IC, Tsai Y-F, Fang J-T, Yeh M-M, Fang J-Y, Liu C-Y. Effects of mouthwash interventions on xerostomia and unstimulated whole saliva flow rate among hemodialysis patients: A randomized controlled study. International Journal of Nursing Studies. 2016;63:9-17.
57. Saeedi M, Morteza-Semnani K, Ghoreishi MR. The treatment of atopic dermatitis with licorice gel. Journal of Dermatological Treatment. 2003;14(3):153-7.
58. Sulzberger M, Worthmann AC, Holtzmann U, Buck B, Jung K, Schoelermann A, et al. Effective treatment for sensitive skin: 4-t-butylcyclohexanol and licochalcone A. Journal of the European Academy of Dermatology and Venereology. 2016;30:9-17.
59. Mou K, Han D, Liu W, Li P. Combination therapy of orally administered glycyrrhizin and UVB improved active-stage generalized vitiligo. Brazilian Journal of Medical and Biological Research. 2016;49(8).
60. Martin MD, Sherman J, Van Der Ven P, Burgess J. A controlled trial of a dissolving oral patch concerning glycyrrhiza (licorice) herbal extract for the treatment of aphthous ulcers. General Dentistry. 2008;56(2):206-10.

جدول ۱. تقسیم بندی بیماران مبتلا به COVID-19

	Disease category	Definition(s)
1	Mild	The clinical symptoms are mild and no pneumonia on imaging
2	Moderate	With fever, respiratory tract and other symptoms, imaging shows pneumonia
3	Severe	Meet any of the following:<br>(a) Shortness of breath, RR> 30 times/minute<br>(b) Finger oxygen saturation is < 93% at rest<br>(c) Arterial blood oxygen partial pressure (PaO2)/oxygen concentration (FiO2) < 300mmHg (l mmHg = 0.133 kPa)<br>At high altitudes (above 1000 meters), PaO2/FiO2 should be corrected according to the following formula: PaO2/FiO2 X [Atmospheric pressure (mmHg)/760]
4	Critical	One of the following:<br>(a) Respiratory failure and requires mechanical ventilation<br>(b) Shock<br>(c) Combining other organ failures requires ICU monitoring and treatment
